# Characterization of a Prophage-Free Derivative Strain of *Lactococcus lactis* ssp. *lactis* IL1403 Reveals the Importance of Prophages for Phenotypic Plasticity of the Host

**DOI:** 10.3389/fmicb.2018.02032

**Published:** 2018-08-31

**Authors:** Anne Aucouturier, Florian Chain, Philippe Langella, Elena Bidnenko

**Affiliations:** MICALIS Institute, INRA, AgroParisTech, Université Paris-Saclay, Jouy-en-Josas, France

**Keywords:** *Lactococcus lactis* IL1403, prophages, prophage impact, prophage-cured strain, physiology of Lactococcus lactis

## Abstract

*Lactococcus lactis* is a lactic acid bacterium of major importance for the dairy industry and for human health. Recent sequencing surveys of this species have provided evidence that all lactococcal genomes contain prophages and prophage-like elements. The prophage-related sequences encompass up to 10% of the bacterial chromosomes and thus contribute significantly to the genetic diversity of lactococci. However, the impact of these resident prophages on the physiology of *L. lactis* is presently unknown. The genome of the first sequenced prototype strain, *L. lactis* ssp. *lactis* IL1403, contains six prophage-like elements which together represent 6.7% of the IL1403 chromosome. Diverse prophage genes other than those encoding phage repressors have been shown to be expressed in lysogenic conditions, suggesting that prophage genes are indeed able to modulate the physiology of their host. To elucidate the effect of resident prophages on the behavior of *L. lactis* in different growth conditions, we constructed and characterized, for the first time, a derivative strain of IL1403 that is prophage-free. This strain provides unique experimental opportunities for the study of different aspects of lactococcal physiology using the well-defined genetic background of IL1403. Here, we show that resident prophages modify the growth and survival of the host strain to a considerable extent in different conditions, including in the gastrointestinal environment. They also may affect cellular autolytic properties and the host cells’ susceptibility to virulent bacteriophages and antimicrobial agents. It thus appears that prophages contribute significantly to lactococcal cell physiology and might play an important role in the adaptation of *L. lactis* to cultivation and environmental conditions.

## Introduction

Lactococci (*Lactococcus lactis* species) are widely used in the industrial manufacturing of fermented dairy products (Leroy and De Vuyst, 2004; Smit et al., 2005). *L. lactis* is also of particular interest for its ever-growing therapeutic and epidemiological applications in the domains of human and animal health (Bermudez-Humaran et al., 2013). For many years, lactococci were an object of intensive fundamental and applied studies, which now have progressed to the fields of “omics-” and systems biology (de Vos, 2011; Kok et al., 2017). Currently, 34 complete and 97 partially assembled lactococcal genomes are available in NCBI’s GenBank^1^. In each one, the genomes of temperate bacteriophages (complete prophages) and prophage remnants have been identified (Chopin et al., 2001; Ventura et al., 2007; Kelleher et al., 2018). Prophage-related sequences encompass from 3 to 10% of the total genome, and represent a significant part of the observed genomic differences among *L. lactis* strains (Kelleher et al., 2018). Genome sequence analyses of lactococcal prophages indicated that they are affiliated with the P335 group of phages, one of the three dominant phage species commonly found in dairy factories (Deveau et al., 2006). The two other species (c2- and 936-like) are composed of phages that are exclusively virulent (Deveau et al., 2006).

The impact of prophages on cell physiology and bacterial ecology has mainly been studied in *Escherichia coli* (Wang et al., 2010) and some bacterial pathogens (reviewed in Fortier and Sekulovic, 2013). Although resident prophages are able to provoke the destruction of a cell, there is a growing appreciation for the beneficial effects they can have for a host. Specifically, there is evidence that prophages can increase host fitness under specific environmental conditions, improve its resistance against infecting phages, or modify cellular metabolism by introducing novel functions or altering pre-existing ones (Wang et al., 2010; Fortier and Sekulovic, 2013; DeBardeleben et al., 2014; Howard-Varona et al., 2017). However, questions about the overall impact of prophages on different aspects of *L. lactis* physiology have not yet been completely resolved. The main reason for this is the lack of an experimental model appropriate for this type of study, namely, a prophage-free strain of *L. lactis* in which all phage-related chromosomal elements have been removed. Progress has been made toward this goal, but the strains generated to date have several drawbacks. For example, a putatively prophage-cured derivative of *L. lactis* ssp. *cremoris* UC509 (UC509.9) still carries a remnant prophage (Ainsworth et al., 2013; Kelleher et al., 2018). Likewise, the derivative strains have been produced of *L. lactis* IL1403, but these still contain three prophage remnants (Visweswaran et al., 2017). Finally, a prophage-free derivative of *L. lactis* ssp. *cremoris* NZ9000 was generated, but in the process several non-essential host genes were also deleted (Zhu et al., 2017).

*Lactococcus lactis* subsp. *lactis* IL1403 strain is the first completely sequenced lactococcal strain (Bolotin et al., 2001). It has been widely used for both fundamental and applied research, including diverse studies of lactococcal genetics and physiology, as well as lactococcal phage biology (Chopin et al., 2005; Douillard and De Vos, 2014; Kok et al., 2017). The genome of IL1403 contains six prophage-like elements, which represent in total 6.7% of the IL1403 chromosome (Chopin et al., 2001). Transcriptional analyses of IL1403 and other lactococcal strains have revealed that, in addition to the genes encoding repressor proteins, other phage-specific genes are expressed in the lysogenic state under different growth conditions (Boyce et al., 1995; Blatny et al., 2003; Seegers et al., 2004; Xie et al., 2004; Cretenet et al., 2011; Dijkstra et al., 2016; Visweswaran et al., 2017). As yet, though, it is unknown if or how these prophage-encoded proteins affect the physiology of the lactococcal cell.

Here, we describe the construction and initial characterization of a derivative of *L. lactis* IL1403 strain in which we deleted all prophage-like elements. We then examined the impact of IL1403 prophages on aspects of cell physiology that are of particular relevance for the industrial and health applications of *L. lactis*. Our results reinforce previous findings of the complex effect of prophages on the behavior of the bacterial cell, and demonstrate the significant contributions of prophages to the adaptive phenotypic plasticity and fitness of *L. lactis*.

## Materials and Methods

### Bacterial Strains, Plasmids, Phages, and Media

*Lactococcus lactis* ssp. *lactis* IL1403, IL1946 (Chopin et al., 1989; Bolotin et al., 2001), and derivative strains were grown at one of two defined temperatures (30°C or 37°C, specified for each experiment) in M17 medium supplemented with 0.5% glucose (M17glu). *E. coli* strain TG1 was grown at 37°C in LB medium. When needed, ampicillin (Ap; 100 μg/ml for *E. coli*), tetracycline (Tc; 5 μg/ml for *L. lactis* and 10 μg/ml for *E. coli*), chloramphenicol (Cm; 10 μg/ml), and erythromycin (5 μg/ml for *L. lactis*) were added to the culture medium. Heme (Sigma) stock solution (0.5 mg/ml) was freshly prepared in alkaline water (0.05 N NaOH). Plasmid pIL1237 is a derivative of pBluscript II SK+ that carries the Tc^R^ gene of pAT182 (Rasmussen et al., 1994), while plasmid pIL253 and pIL253 derivative, which carry Cm^*R*^ gene, were from our laboratory collection. Reference prolate phages (c2, bIL67), small isometric-headed phages (sk1, bIL66, bIL170, bIL41), and phages isolated from industrial samples (bIL8, bIL10, bIL13, bIL14, bIL19, bIL20, bIL32, and bIL39) were also from our laboratory collection. Phages were sequentially propagated in suitable *L. lactis* hosts and, when required, in *L. lactis* IL1403 or *L. lactis* IL6288 strains using M17glu medium supplemented with 10 mM CaCl_2._ Phages were enumerated as described in (Anba et al., 1995).

### Measurement of Growth Rate and Cell Survival

For growth-rate experiments, *L. lactis* strains were incubated overnight in M17glu medium at 30°C in static (non-aerated) conditions, then diluted to OD_600_ 0.05 (measured with a Novaspec II Visible Spectrophotometer, Pharmacia Biotech) in fresh M17glu medium that contained, when required, different concentrations of heme (Sigma), from 0 to 100 μg/ml. We then transferred 200 μl of each sample to a 96-well plate. All plates (with the lids on to avoid evaporation) were incubated at the temperature indicated for each experiment, with or without constant shaking, in a multimode microplate reader (Synergy 2, BioTek Instruments, Inc) for the time indicated. The OD_600_ was measured at 15-min intervals; the static culture was gently agitated before each measurement. Growth experiments were repeated at least four times, and each experiment included four independent cultures of each strain. For the survival experiments, time and temperature conditions are specified in the Results and Discussion section, but the general procedure was as follows. Bacterial cultures were grown overnight in M17glu medium, then diluted in fresh medium. Static cultures were grown without agitation (culture volume 20 ml in 50-ml bottle) at 30°C or 37°C. To determine cell viability, aliquots were taken at different time points, serially diluted in Ringer’s saline solution (Merck, Germany), and plated on M17glu agar. Colony-forming units (CFUs) were counted after 36 h of incubation at 30°C. Each data point (CFU/ml) was the mean of the counts of at least three independent cultures.

### Lysozyme and Nisin Susceptibility Assays

The susceptibility of *L. lactis* to antimicrobials was determined using M17 agar plates. Overnight cultures of IL1403 and its derivative strains were diluted 10-fold in Ringer’s saline solution (Merck). Drops (5 μl) of the dilutions were spotted on M17glu agar that contained different concentrations of inhibitors: from 0 to 0.5 mg/ml of lysozyme (Fluka) and from 0 to 300 ng/ml of nisin (Sigma). Plate counts were performed after 36 h of incubation at 30°C. The experiment was reproduced three times.

### Plate Test of Autolytic Activity

Lytic activity of growing *L. lactis* cells was examined by plating the diluted overnight cultures on M17 agar plates that contained 0.2% (wt/vol) autoclaved, lyophilized cells of *Micrococcus luteus* ATCC 4698 (Sigma). Plates were incubated at the indicated temperatures for 48 h and then examined for the appearance of halos around the lactococcal colonies. The experiment was performed three times. Images were acquired with a ChemiDoc MP system (Bio-Rad).

### Autolysis in Buffer Solution

*Lactococcus lactis* strains were grown in M17 medium to an OD_600_ of 0.6. Cells were harvested by centrifugation at 5000 *g* for 15 min at 4°C, washed once with sterile 50 mM potassium phosphate (PBS) buffer (pH 7.0), and resuspended in the same buffer to OD_600_ 0.7; supplemented when required with 0.05% Triton X-100 (Bio-Rad). Cell suspensions were transferred into 100-well sterile microplates and incubated at 30°C or 37°C. Autolysis was monitored by measuring the OD_600_ of the cell suspensions at 15-min intervals with an automated multi-mode plate reader (Synergy 2, BioTek Instruments, Inc). This experiment was performed twice. Each experiment included four independent cultures of each strain. The extent of autolysis was expressed as the percentage decrease in OD_600_.

### Heme-Induced Toxicity Assay

Autoclaved heme (Fluka) was added to exponentially growing cultures of *L. lactis* IL1403 and IL6288 (OD_600_ of 0.6) at concentrations ranging from 0 to 50 μg/ml. Cultures were incubated at 30°C under non-aerated conditions. After 1 h, the number of viable cells was determined by diluting the cultures in Ringer’s saline solution and spotting 3 μl onto M17glu agar. Images were acquired with a ChemiDoc MP system after 36 h of incubation at 30°C. This experiment was performed twice.

### Molecular Cloning and DNA Sequence Analysis

The procedures used here for DNA manipulation, cloning, and transformation of *E. coli* and *L. lactis* were carried out, for the most part, as described in (Sambrook et al., 1989; Anba et al., 1995). Polymerase chain reactions (PCRs) were performed using the Gene AMP PCR System 9700 thermal cycler (Applied Biosystems) and ExTaq (Takara Biomedicals) essentially as recommended by the supplier. Nucleotide sequencing was performed on PCR products using appropriate primers (**Supplementary Table S1**), Taq polymerase (Applied Biosystems), and fluorescent dideoxyribonucleotides on a 377A DNA Sequencer (Applied Biosystems).

### Curing of Resident Prophages From IL1403 Chromosome

First, we constructed plasmids that carried DNA fragments corresponding to the early genome regions of different IL1403 prophages; this was performed by cloning *SmaI*-digested phage-specific DNA into the *Eco*RV site of the pIL1237 plasmid vector. DNA fragments used for cloning were amplified using the IL1403 chromosome as a template and specific oligonucleotides (AA07/AA08 for bIL309; AA30/AA31 for bIL286; AA32/AA33 for bIL310; AA34/AA35 for bIL312) that contained *SmaI* sites (see **Supplementary Table S1** for oligonucleotides used in this study). Cloning was performed in *E. coli* strain TG1. The resulting plasmids were designated pIL1237::bIL309, pIL1237::bIL286, pIL1237::bIL310, and pIL1237::bIL312.

To delete bIL309 prophage, we started with the previously constructed bIL285-free strain IL1946 (Chopin et al., 1989) and transformed it with the pIL1237::bIL309 plasmid. Five Tc^R^ colonies were tested by PCR to verify the correct integration of pIL1237 at the bIL309, using oligonucleotide pairs AA26/MCC31, AA27/MCC32, and AA26/AA27. One PCR-positive clone was selected for induction experiments. To induce prophage excision, cells were grown to OD_600_ 0.6, serial dilutions were spotted on M17 agar plates, and plates were irradiated with UV light (at 254 nm) with an energy of 40 J/m^2^ for 2–4 s with a Stratalinker 2400 UV source (Stratagene). Next, 24 Tc^S^ clones selected after irradiation were tested by PCR with the oligonucleotide pair AA26/AA27 to detect the excision of bIL309; 4 PCR-positive clones were subsequently verified by sequencing. The resulting *L. lactis* strain (bIL285- and bIL309-free) was named IL6248 (**Table 1**).

**Table 1:** Prophage-free derivative strains of *L. lactis* IL1403.

Strain	Parental strain	Resident prophages					
IL1403		bIL285	bIL286	bIL309	bIL310	bIL311	bIL312
IL1946	IL1403		bIL286	bIL309	bIL310	bIL311	bIL312
IL6248	IL1946		bIL286		bIL310	bIL311	bIL312
IL6250	IL6248				bIL310	bIL311	bIL312
IL6254	IL6250					bIL311	bIL312
IL6260	IL6250					bIL311	bIL312
IL6277	IL6260						bIL312
IL6288	IL6277						
IL6345	IL1403	bIL285		bIL309	bIL310	bIL311	bIL312
IL6353	IL6345	bIL285			bIL310	bIL311	bIL312
IL6351	IL1946			bIL309	bIL310	bIL311	bIL312
IL6328	IL6260					bIL311	
IL6354	IL6288		bIL286				


Next, to delete bIL286 prophage from this strain, IL6248 cells were transformed with the pIL1237::bIL286 plasmid. Seven IL6248 Tc^R^ colonies were tested by PCR for chromosomal integration using the oligonucleotide pairs AA28/AA41, AA30/AA29, AA39/AA40, AA37/AA40, AA36/AA37, and AA36/AA39. One PCR-positive clone was used for induction experiments. Induction was performed by irradiation with UV light (254 nm) with an energy of 60 J/m^2^ for 2–4 s. Verification of the prophage-free derivative strain was performed by PCR using the oligonucleotide pair AA28/AA29. The resulting *L. lactis* strain (bIL285-, bIL309-, and bIL286-free) was named IL6250 (**Table 1**).

To delete bIL310 prophage, strain IL6250 was transformed with the pIL1237::bIL310 plasmid. Seven IL6250 Tc^R^ colonies were tested by PCR for chromosomal integration at the bIL310 using the oligonucleotide pairs MCC35/AA41, MCC44/AA42, and AA41/AA42. One PCR-positive clone was used for induction experiments. Induction was performed by irradiation with UV light (254 nm) with an energy of 60 J/m^2^ for 2–4 s. Verification of the prophage-free derivative strain was performed as described above. Two clones of the resulting *L. lactis* strain (bIL285-, bIL309-, bIL286-, and bIL310-free) were named IL6254 and IL6260 (**Table 1**). To delete bIL312 prophage, strain IL6254 was transformed with the pIL1237::bIL312 plasmid, but all efforts to obtain Tc^R^ integrants were unsuccessful. We then checked for the presence of bIL312 in strains IL6254 and IL6260 (both bIL285-, bIL309-, bIL286-, bIL310-free) using the oligonucleotide pairs AA43/AA44, AA43/AA56, AA34/AA35, AA44/AA37, and AA56/AA57; we wanted to identify clones in which bIL312 had been spontaneously lost as the result of the previous treatment with UV light. Among approximately 1000 clones, three clones derived from IL6260 did not carry bIL312. The verification of the selected clones was performed as described above. The resulting *L. lactis* strain (bIL285-, bIL309-, bIL286-, bIL301-, and bIL312-free) was named IL6328 (**Table 1**).

A bIL311-free strain was obtained with the use of the pGhost9 plasmid integration mutagenesis system (Maguin et al., 1996). DNA fragments that corresponded to the early region of the bIL311 genome were amplified using the oligonucleotide pairs AA53/AA60 and AA47/AA58. They were digested with *SmaI* and *Kpn*I and cloned into pGhost9 in *E. coli* TG1 cells. Next, the resulting plasmid was introduced into IL6260 strain. Selection of integrants was performed after shifting the temperature to 37.5°C and cells plating on M17glu agar supplemented with Em. The integration was confirmed by PCR amplification. Excision of the integrated pGhost construct, which led to the deletion of bIL311, was performed by shifting the temperature to 30°C and selecting for Em^S^ clones. The resulting *L. lactis* strain (bIL285-, bIL309-, bIL286, bIL310, and bIL311-free) was named IL6277 (**Table 1**). Deletion of bIL312 prophage from IL6277 was performed via transformation with the pIL1237::bIL312 plasmid, the selection of four Tc^R^ integrants, and consequent induction of the prophage by irradiation with UV light (254 nm) with an energy of 60 J/m^2^ for 2–4 s. The resulting, completely prophage-free, strain IL1403 (bIL285-, bIL309-, bIL286-, bIL301-, bIL312-, and bIL311-free) was verified by sequencing and named IL6288 (**Table 1**). Strain IL6351 (bIL285-, bIL286-free) was constructed from IL1946 using the pIL1237::bIL286 plasmid essentially as described above (**Table 1**). In addition, bIL286 was deleted from IL1403 strain as described above, the resulting strain was named IL6345 (**Table 1**). Next, IL6353 (bIL286, bIL309-free) stain was constructed from IL6345 by the constitutive insertion and induction of pIL1237::bIL309 plasmid.

Finally, we constructed the lysogenic strain IL6354 by re-introducing bIL286 into the prophage-free strain IL6288; this was performed using the superinfection plate assay with total phage lysate as described in (Reyrolle et al., 1982). Briefly, phage lysate was prepared from 10 ml of exponentially growing IL1403 culture treated with mitomycin C (final concentration of 0.5 μg/ml/) at 30°C. Lysis was detected by measuring the OD_600_ for 4 h at 30-min intervals following the addition of mitomycin C. Cellular debris was removed by centrifugation at 8,000 rpm for 10 min at 4°C. Between 10 and 20 μl of the lysate were immediately spotted on M17glu agar plates in which the top agar had been mixed with IL6288 cells. A non-treated culture served as the control. Plates were incubated at 30°C for 36 h. The individual colonies that grew within the lysis zones were examined for the presence of the prophage using PCR with the oligonucleotide pairs mentioned above and subsequent sequencing. One clone carried the bIL286 prophage at its initial location; it was retained and named IL6354 (**Table 1**).

### Mouse Experiment

Germ-free mice (8-weeks-old, C3H) were provided by ANAXEM, the germ-free rodent breeding facility of the MICALIS institute (INRA, Jouy-en-Josas, France). All animals were housed in flexible-film isolators (Getinge-La Calhène, Vendôme, France). Three isolators were used; each contained one cage of four mice. Mice were provided, *ad libitum*, sterile tap water and a gamma-irradiated standard diet (R03-40, S.A.F.E., Augy, France). Their bedding was composed of wood shavings and they were also given cellulose sheets as enrichment. The light/dark cycle was 12 h/12 h, the temperature was maintained between 20 and 22°C, and humidity was between 45 to 55%. All procedures related to the use of germ-free mice were approved by the Local Ethics Committee (Comethea: reference number 45) and by the French Ministry of Research, and were recorded under the project number 3441-2016010614307552. Once a day for 3 consecutive days, mice were orally gavaged with 16% glycerol solution in PBS buffer that contained either 10^9^ CFUs of strain IL1403, or 10^9^ CFUs of strain IL6288, or the mixture of IL1403 (pIl253:Em^R^) and IL6288 (pIL253: Cm^R^) strains (10^9^ CFUs of each strain). Feces were collected and weighed at the indicated day after gavage. Then, feces were resuspended, vortexed and diluted in PBS buffer. The survival and stability of *L. lactis* was checked by plating fecal material on M17glu agar either antibiotic-free or supplemented with Em or Cm for the detection of IL1403 or IL6288, respectively, in the pure or the mixed cultures. CFUs were counted after 36 h of anaerobic cultivation at 30°C.

## Results and Discussion

### Construction of Prophage-Free Derivatives of *L. lactis* IL1403

**FIGURE 1:**
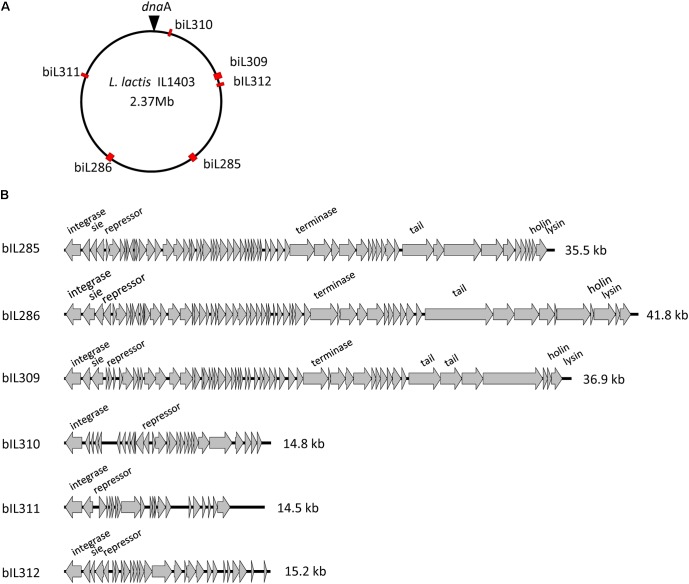
*L. lactis* IL1403 prophages. **(A)** Location of prophages on the chromosome of *L. lactis* IL1403. The prophage positions are marked by red rectangles; the position of *dnaA* within the *oriC* region is designated by a triangle. **(B)** Genome organization of the *L. lactis* IL1403 prophages. Genome boundaries correspond to *attB* sites. Genes are represented by arrows oriented in the direction of transcription. Functions of selected early- and late-gene products are indicated.

As was described previously (Bolotin et al., 2001; Chopin et al., 2001), the genome of *L. lactis* strain IL1403 contains six randomly distributed prophage-like elements: bIL285 (35538 bp), bIL286 (41834 bp), bIL309 (36949 bp), bIL310 (14957 bp), bIL311 (14510 bp), and bIL312 (15179 bp) (**Figure 1A**). The bIL309 is integrated into a tRNA^arg^ gene. Five other prophages are integrated into non-coding regions of the IL1403 chromosome. The bIL312 is integrated between the tRNA^met^ gene and the *hslA* gene, which codes for the bacterial histone-like protein HU and is transcribed in the opposite direction of the phage integrase gene. The bIL285 is integrated between the *ykhD* gene (codes for redox-sensing transcriptional regulator) and the *radC* gene (codes for DNA repair protein), which are oriented in the opposite direction to the prophage lysin and integrase genes, respectively. The bIL286 is integrated between the tRNA^ser^ gene and the *yofM* gene. This latter gene is transcribed in the opposite direction of the phage integrase gene and it appears to be similar to the *ylxM* gene of *Bacillus subtilis*, which encodes a component of a putative signal recognition particle. Prophage bIL310 is integrated between the genes *yofE* and *mtlD*, which encode, respectively, a protein of unknown function and mannitol-1-phosphate 5-dehydrogenase. Finally, prophage bIL311, which carries two IS983 elements within its early-genes region, is located between the *yucF* and *pdc* genes, which code for a protein of unknown function and phenolic acid decarboxylase, respectively.

Prophage-like elements can be divided into two groups: the first comprising the three prophages with large genomes (33–42 kb; bIL285, bIL286, bIL309) and the second composed of the three prophages with small genomes (14–15 kb; bIL310, bIL311, and bIL312) (**Figure 1B**). The three smaller IL1403 prophages appear to lack the genes required for phage morphogenesis and lysis of the host cell; they have thus been considered to be prophage remnant elements. It was reported previously that the repression system that maintains lactococcal prophages in the lysogenic state in different lactococcal strains is quite tight: prophages do not appear to be activated by individual stressors such as low or high temperatures, high osmotic pressure, hydrochloric or lactic acids, hydrogen peroxide, or the antibiotic bacitracin, which targets peptidoglycan synthesis (Xie et al., 2004; Ho et al., 2016). However, treatment with ultraviolet (UV) light does cause the induction of all IL1403 prophages with the exception of bIL311 (Chopin et al., 2001), a finding that was previously employed for the deletion of the bIL285 prophage from IL1403 strain (Chopin et al., 1989). We used this existing bIL285-free strain (IL1946) and eliminated four of the remaining prophages (bIL286, bIL309, bIL310, and bIL312) via the construction of single-copy integrants using the non-replicative pIL1237 plasmid that carried the Tc^R^ gene. Prophage genomes that were marked with Tc^R^ gene were induced by UV light to enable the selection of Tc^S^ prophage-free clones (see the Section “Materials and Methods”). However, this method was not successful in deleting the bIL311 prophage, which was subsequently accomplished using the pGhost9 plasmid integration mutagenesis system (Maguin et al., 1996) (see the Section “Materials and Methods”). The final prophage-free derivative of strain IL1403, cured of all six prophages, was designated IL6288. In addition, we constructed several intermediate IL1403 derivatives that contained different combinations of prophages. The IL6345 and IL6353 strains were constructed by the successive deletions of bIL286 and bIL309 prophages from the parental IL1403 strain. We also evaluated the ability of the prophage-free strain, IL6288, to serve as a potential host in re-lysogenization experiments that used total phage lysate obtained after the induction of lysogenic strain IL1403 (see the Section “Materials and Methods”). Using this approach, we found that only phage bIL286 was able to re-lysogenize the prophage-free derivative strain; this was consistent with a previous suggestion that all the other prophages are defective (Chopin et al., 2001). The relevant genotypes of all constructed strains are listed in **Table 1**.

### Resident Prophages Modify the Susceptibility of Host Cells to Virulent Phages

The presence of prophages in a bacterial chromosome alters the sensitivity of the host strain to infecting phages by different mechanisms. Constitutive expression of prophage-encoded repressor proteins ensures immunity of the host to infection with related temperate phages. Two other mechanisms, the abortive infection (Abi) and superinfection exclusion (Sie) resistance systems protect bacteria from virulent phages (Chopin et al., 2005; Labrie et al., 2010). Strain IL1403 does not possess any Abi system, but four prophage-encoded *sie* genes have been formally identified in its genome: *orf*2 (*sie*_285_) in bIL285, *orf2* (*orf2*_286_), in bIL286, *orf2* (*sie*_309_) in bIL309, and *orf2* (*sie*_312_) in bIL312 (McGrath et al., 2002; Mahony et al., 2008). These *sie* genes are located within the phage lysogeny modules; they are expressed in the lysogenic state and encode proteins with membrane-spanning regions and/or a hydrophobic N-terminus (McGrath et al., 2002; Mahony et al., 2008). When produced using the nisin-inducible expression system in *L. lactis* ssp. *cremoris* strain MG1363, *sie*_309_ and *sie*_312_ provided resistance against several phages from the 936 phage group by blocking phage DNA injection. Sie proteins have been detected in different IL1403 proteomes, indicating that these proteins are produced in substantial amounts (Drews et al., 2004; Beganovic et al., 2010).

To examine the impact of resident prophages on a cell’s sensitivity to infection with virulent phages, *L. lactis* IL1403 and its derivative strains were assayed against a range of 936- and c2-like phages (see the Section “Materials and Methods”). For 12 of the 14 virulent phages from our laboratory collection, phage titers and plaque morphology were identical for all tested strains (**Table 2**, data shown for bIL170 and bIL67). However, the presence or absence of resident prophages had a detectable effect on the activity of phages bIL14 and c2; these two virulent phages had different titers and showed variable plaque morphology when propagated on the parent strain IL1403 or its prophage-free derivative IL6288 (**Table 2**). Both properties were unaffected by the identity of the host strain (IL1403 or IL6288) that had been used for initial phage propagation (data shown for phage stocks prepared in IL1403 strain).

**Table 2:** Resident prophages of *L. lactis* IL1403 interfere with infection by virulent phages.

Strain	Relevant genotype						Phage titer*^a^ (PFU/ml)*^b^**			
							936 Phage species		c2 Phage species	
							bIL170	bIL14	bIL67	c2
IL1403	bIL285	bIL286	bIL309	bIL310	bIL311	bIL312	1 × 10^8^ c*^c^*	2.7 × 10^6^ c; 1.2 × 10^6^ t*^d^*	1 × 10^8^ c	1.1 × 10^5^ c, s*^e^*; 3.3 × 10^6^ t
IL1946		bIL286	bIL309	bIL310	bIL311	bIL312	1 × 10^8^ c	8.4 × 10^5^ c; 8 × 10^5^	1 × 10^8^ c	2.5 × 10^4^ c, s; 5.1 × 10^4^ t
IL6345	bIL285		bIL309	bIL310	bIL311	bIL312	1 × 10^8^ c	4.4 × 10^7^ c	1 × 10^8^ c	2.5 × 10^5^ c, s; 2.6 × 16^5^ t
IL6351			bIL309	bIL310	bIL311	bIL312	1 × 10^8^ c	2 × 10^5^ c	1 × 10^8^ c	1.8 × 10^3^ c, s; 9.1 × 10^4^
IL6248		bIL286		bIL310	bIL311	bIL312	1 × 10^8^ c	4.1 × 10^5^ c	1 × 10^8^ c	1 × 10^5^ c, s; 2.2 × 10^5^ t
IL6353	bIL285			bIL310	bIL311	bIL312	1 × 10^8^ c	1 × 10^6^ c; 6 × 10^5^ t	1 × 10^8^ c	2.3 × 10^5^ c, s; 1.5 × 10^6^ t
IL6250				bIL310	bIL311	bIL312	1 × 10^8^ c	4.5 × 10^7^ c	1 × 10^8^ c	8.9 × 10^4^ c, s; 7.2 × 10^4^ t
IL6260					bIL311	bIL312	1 × 10^8^ c	1.5 × 10^6^ c; 5 × 10^6^ t	1 × 10^8^ c	3 × 10^4^ c, s; 3 × 10^5^ t
IL6328					bIL311		1 × 10^8^ c	2.1 × 10^6^ c; 3.8 × 10^6^ t	1 × 10^8^ c	4 × 10^5^c; 2 × 10^5^t
IL6277						bIL312	1 × 10^8^ c	1.8 × 10^6^ c; 5.1 × 10^6^ t	1 × 10^8^ c	1 × 10^6^ c; 6 × 10^6^ t
IL6354		bIL286					1 × 10^8^ c	4.5 × 10^7^ c	1 × 10^8^ c	9.5 × 10^6^ c; 1 × 10^6^ t
IL6288	Prophage-free						1 × 10^8^ c	4.9 × 10^7^ c	1 × 10^8^ c	8.8 × 10^6^ c; 1.5 × 10^7^ t

*a, average of three independent experiments; *b*, PFU: plaque forming units; *c*, c: clear plaques; *d*, t: turbid plaques; *e*, s: small plaques*.

**FIGURE 2:**
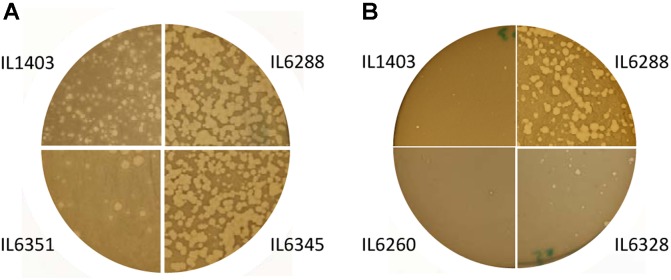
Morphology of the lysis plaques formed by **(A)** the 936-like small isometric-headed phage bIL14 and **(B)** the prolate-headed phage c2 in the parental strain IL1403 and its derivative strains: IL6351 (bIL285-, bIL286-free), IL6345 (bIL286-free), IL6260 (bIL285-, bIL286-, bIL309-, bIL310-free), IL6328 (bIL285-, bIL286-, bIL309-, bIL310-, bIL312-free), and IL6288 (prophage-free). The experiment was reproduced at least three times. The results shown are from a representative experiment.

The development of the 936-like phage bIL14 was positively affected by the deletion of all six prophages: the titer of bIL14 obtained by plating on IL6288 was more than ten times higher than on the parental strain (**Table 2**). Additionally, no turbid plaques were detected on strain IL6288 (**Figure 2A**). Instead, bIL14 appeared to have varying effects on the intermediate strains. The development of bIL14 in strains IL1946, IL6353, IL6260, IL6328, and IL6277 was similar to that in IL1403, while the development of bIL14 in IL6250, IL6345, and IL6354 was similar to the prophage-free strain (**Figure 2A** and **Table 2**). Thus, it appeared that bIL14 had the maximal plating efficiency on strains, which were deleted for bIL286 either singly (as in strain IL6345) or together with other large prophages (IL6250). This could be evidence that the bIL286-encoded *orf2*_286_ *sie* gene plays an important role in bIL14 infection. However, the stimulatory effect of bIL286 deletion on the efficiency of bIL14 infection disappeared with the single deletion of either bIL285 or bIL309 (IL6351 and IL6353, respectively, **Table 2**), which suggests that the relationship between the three prophages is complex. Furthermore, the simultaneous deletion of the three small prophages (bIL310, bIL311, and bIL312) increased bIL14 plating efficiency to the level found in the prophage-free strain, even in the presence of bIL286 (IL6354). Instead, the intermediate strains IL6248 and IL6351 showed lower sensitivity to bIL14 than the parental strain did (**Figure 2A**). These strains carry all three small prophages (bIL310, bIL311, bIL312) and either bIL286 or bIL309, but lack bIL285. At present, the molecular basis for the behavior of the bIL14 phage cannot be explained because its genome sequence is unknown. However, these results are consistent with previously reported data that indicated that prophage-encoded Sie defense systems were effective only against a limited group of the virulent 936-like phages, based on a highly specific direct interaction between the Sie protein and a structural element of the adsorbed phage (Mahony et al., 2008).

For the prolate-headed phage c2, the highest titers and clear plaques were likewise found in the prophage-deleted strain, IL6288, while the lowest titers and the mixture of small clear and turbid plaques were found in IL1403 and any strain carrying the two small prophages bIL311 and bIL312 (i.e., strains IL6250, IL1946, IL6248, IL6345, IL6351, IL6353, and IL6360) (**Figure 2B** and **Table 2**). The presence of phage bIL311 played a major role in the inhibition of c2 infection (compare strains IL6328 and IL6277, **Table 2**), but this inhibitory effect was further intensified by the presence of bIL312 (compare strains IL6328 and IL6260, **Figure 2B** and **Table 2**). Previous investigations of the bIL312 phage found that its *orf2* (*sie*_312_) gene provided resistance against 936-like phages, but not c2 phages; instead, no putative resistance genes have yet been identified in the genome of bIL311 (McGrath et al., 2002; Mahony et al., 2008). This suggests that the effects observed here were caused by as-yet-unidentified bIL311- and bIL312-encoded resistance function(s). It has been shown recently that two late bIL311 genes, *orf17* and *orf18*, and one bIL312 gene, *orf21*, are expressed in the host IL1403 strain (Dijkstra et al., 2016). The predicted Orf17 protein shows homology to proteins from the GNAT family of acetyltransferases. The predicted Orf18 protein contains a helix-turn-helix DNA-binding motif. No homology to proteins of known function can be detected for bIL312 *orf21*.

It should be noted that the genomes of two prolate-headed phages tested here, bIL67 and c2, are highly similar at the overall nucleotide level, but there is a region that is much less conserved (51.9% of identity) which contains three late-expressed genes involved in host-range determination (Lubbers et al., 1995; Millen and Romero, 2016). To infect a host cell, all c2-like phages first recognize carbohydrate receptors on the cell surface in order to ensure the reversible binding of the phage to the host cell. Next, c2 phage interacts with the cell-membrane protein receptor Pip (phage infection protein), which results in irreversible binding and is essential for subsequent injection of phage DNA (reviewed in Mahony et al., 2016). However, bIL67 requires a different cell-membrane-associated protein receptor, YjaE. The factors that determine the utilization of either Pip or YjaE are encoded by genes from the non-conserved genomic regions of c2 and bIL67, respectively (Millen and Romero, 2016). This may explain the different behavior observed here between c2 and bIL67 phages during infection of the wild-type and prophage-free strains: it could be that the prophage-encoded proteins modify the arrangement of the specific cellular receptors required for infection by c2, but not by bIL67.

Altogether, our results indicate that prophage genes may contribute to the resistance of their host to virulent phage infection. In addition to the *sie* genes previously identified in the IL1403 genome, other as-yet-unidentified prophage-encoded functions (for example, those encoded by bIL311) are probably involved in phage resistance and await further investigation.

### Prophage-Free Strain Shows Increased Resistance to Cell-Wall-Specific Antimicrobials

To compare the susceptibility of IL1403 and its prophage-free derivative to antimicrobial agents, we selected two commonly used cell-wall inhibitors, lysozyme, and nisin. Lysozyme, which is an important element of the innate immune system in animals, acts through hydrolysis of peptidoglycan (PG) sugar chains. In addition to this enzymatic activity, lysozyme, like nisin, also has non-enzymatic inhibitory effects based on cationic antimicrobial peptide (CAMP) activity; this kills bacteria through the formation of pores and destabilization of the cytoplasmic membrane via mechanisms involving interactions with phospholipids (Herbert et al., 2007; Roy et al., 2009). Bacterial resistance to CAMP activity occurs through modification of the cellular membrane or the interception of CAMPs by cell envelope-associated or secreted proteases (Roy et al., 2009; Koprivnjak and Peschel, 2011).

**FIGURE 3:**
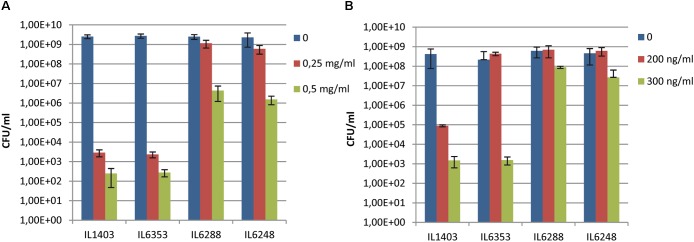
Sensitivity of *L. lactis* IL1403 and its derivative strains IL1946 (bIL285-free), IL6353 (bIL286-, bIL309-free), and IL6288 to **(A)** lysozyme and **(B)** nisin. Serial dilutions of overnight cultures (from 10^-3^ to 10^-5^) were spotted on M17glu agar containing either lysozyme (from 0 to 0.5 mg/ml; **A**) or nisin (from 0 to 300 ng/ml; **B**). Colonies were counted after 36 h of incubation at 30°C. The bars represent average values from three independent experiments, including two biological replicas for each strain.

To test the impact of the resident prophages on their host’s sensitivity to antimicrobial agents, we examined the growth of IL1403 and its derivatives in the presence of lysozyme or nisin (see the Section “Materials and Methods”). As shown in **Figures 3A,B**, parental strain IL1403 was highly sensitive to both lysozyme and nisin, while its prophage-free derivative, IL6288, was much more resistant to both antimicrobials. The IL6353 strain, which carried the bIL285 prophage, showed the same pattern of growth inhibition as IL1403, while the bIL285-free derivatives tested (IL1946, IL6351, IL6248) were similar to IL6288 in showing increased resistance to both antimicrobials (data shown for IL6248).

Our results demonstrated that the resident prophages, especially bIL285, increase the sensitivity of IL1403 to antimicrobials. We suggest that the cause of this may lie in modifications made by prophage-encoded proteins to the bacterial membrane and/or PG that increase host sensitivity to lysozyme and nisin. For example, the *orf2* (*sie*_285_) protein, encoded by the constitutively expressed lysogeny module, could serve as a mediator of the CAMP-membrane interaction. *orf2* (*sie*_285_) was detected in the proteome of *L. lactis* (Drews et al., 2004) and later recognized as a membrane protein that contains a bacterial Pleckstrin-homology (PH*b*) domain (Xu et al., 2010). It has been shown that membrane-associated PH*b*-containing proteins can interact with phosphatidylinositol lipids (Xu et al., 2010). Thus, it may be that the *orf2* (*sie*_285_) protein modifies the phospholipid organization of the membrane and thereby increases host sensitivity to antimicrobials. In addition, the cell-wall hydrolase activity encoded by the bIL285 and/or bIL286 prophages (Visweswaran et al., 2017) may also contribute to fragility of the cell wall and increase the sensitivity of the parent strain to cell-wall-specific antimicrobials. Similarly, an increased sensitivity to lysozyme was recently demonstrated for lysogenic *Streptococcus suis* strain SS2-4 compared to its prophage-free derivative, but no molecular mechanism was proposed to explain this observation (Tang et al., 2013).

The experimental validation of the involvement of phage-encoded lysins and/or *orf2* (*sie*_285_) in the sensitivity of lactococci to antimicrobials requires further investigation.

### Resident Prophage Modifies the Autolytic Properties of *L. lactis* IL1403

The autolytic properties of *L. lactis* are crucial for its use in dairy fermentation and are also considerated in health applications (Lortal and Chapot-Chartier, 2005; Bermudez-Humaran et al., 2013). Bacterial autolysis is a complex phenomenon determined by multiple factors: the composition and structure of cellular PG, the expression and activity of peptidoglycan hydrolases (PGHs) and proteolytic enzymes, and the activity of prophage-encoded lytic enzymes (Lepeuple et al., 1998; O’Sullivan et al., 2000; Pillidge et al., 2002; Lortal and Chapot-Chartier, 2005; Visweswaran et al., 2017). Altogether, these factors determine important variations in autolysis levels that are found among lactococcal strains, and make the selection of an appropriate autolytic strain a difficult task. Previous studies of the autolysis of lactococcal cells have investigated the role of the main cellular autolytic enzyme, *N*-acetyl muramidase (AcmA), and the influence of the prophages bIL285, bIL286, and bIL309, which possess two-component lysis cassettes that encode lysin-holin complexes (Pillidge et al., 2002; Labrie et al., 2004; Visweswaran et al., 2017). Expression of the corresponding prophage genes was not detected by global transcriptome analyses, but was instead demonstrated by qRT-PCR (Xie et al., 2004; Cretenet et al., 2011; Visweswaran et al., 2017). Phage-specific endolysin activity was detected in the cell-free extracts only after UV induction of prophages (Visweswaran et al., 2017).

**FIGURE 4:**
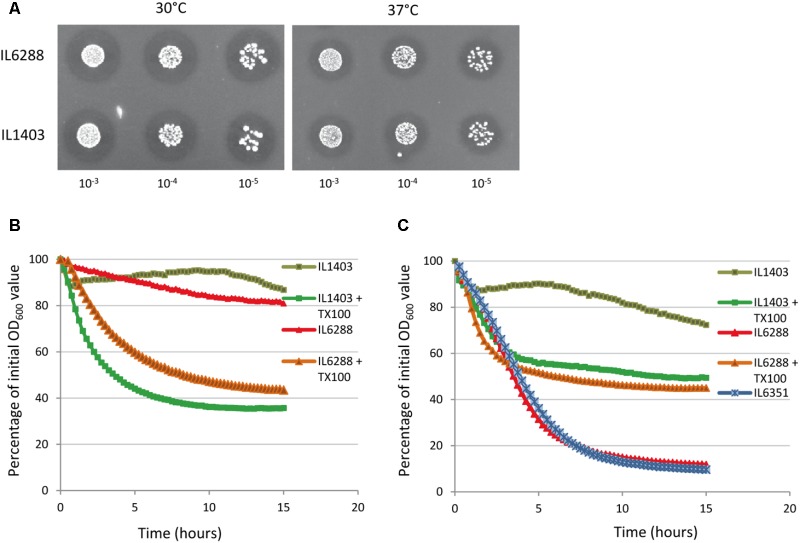
Autolytic properties of IL1403 and its prophage-free derivative strain IL6288. **(A)** Lytic activity of growing *L. lactis* IL1403 and IL6288 cells was examined by plating serial dilutions of overnight cultures on M17glu agar plates that contained 0.2% (wt/vol) autoclaved, lyophilized cells of *M. luteus* ATCC 4698. Images were acquired with a ChemiDoc MP System after 48 h of incubation at 30°C or 37°C. Autolysis of *L. lactis* IL1403 and its derivative strains IL6351 (bIL285-, bIL286-free) and IL6288 (prophage-free) at 30°C **(B)** and 37°C **(C)**. Exponentially grown cells were washed and re-suspended in 50 mM potassium phosphate buffer in the absence or presence of 0.05% Triton X-100 (+TX100). Lysis was monitored by measuring the OD_600_ of the cell suspensions at 15-min intervals with an automated multimode plate reader (Synergy 2, BioTek Instruments, Inc). For each strain, the mean values of three independent suspensions, analyzed simultaneously, are plotted and expressed as the percentage decrease in OD_600_, the SDs were ≤ 10%. Similar autolytic profiles were obtained in three independent experiments. The results presented are of a representative experiment.

To examine the impact of the resident prophages on the autolysis of growing lactococcal cells, we first applied the standard plate assay for the detection of PGH activity, using lyophilized *M. luteus* cells at both 30°C (to simulate fermentation conditions) and at 37°C (to simulate the gastrointestinal environment) (see the Section “Materials and Methods”). We observed no difference between the halos formed by the parent and the derivative strains during growth at either 30°C or 37°C (**Figure 4A**, data shown for IL1403 and IL6288). This suggests that the main cellular autolysin, AcmA, exhibits the same level of activity in all tested strains and that the contribution of the prophage-encoded lytic enzymes to cellular autolytic activity is insignificant in the tested growth conditions. Next, to determine the impact of the resident prophages on autolysis of non-growing cells, we performed a standard turbidimetric assay using autolysis buffer that contained the non-ionic detergent Triton X-100 (see the Section “Materials and Methods”); Triton X-100 permeabilizes bacterial cells via solubilization of integral membrane proteins and does not affect PG composition or the activity of PGHs (Schnaitman, 1971). As shown in **Figures 4B,C**, the prophage-free strain exhibited an autolysis rate similar to that of the parental strain at both 30°C and 37°C. In order to examine the putative roles of the prophage-encoded integral membrane proteins in IL1403, we repeated the autolysis test without the use of Triton X-100. At 30°C, both strains exhibited weak autolytic activity, which is characteristic for non-induced autolysis. Furthermore, IL1403 showed similar autolysis profiles at 37°C in the presence or absence of Triton X-100. Instead, in the absence of the detergent the autolysis rate of IL6288 at 37°C increased significantly compared to that of IL1403 (**Figure 4C**). To identify the prophage(s) primarily responsible for the observed phenotype, we assessed the autolytic properties at 37°C of *L. lactis* IL1403 derivatives that contained different prophages. In the presence of Triton X-100, all showed autolytic profiles identical to the parental strain. However, in the absence of Triton X-100, profiles differed among strains. Strains IL6351 and IL6248 (both lacking bIL285) showed autolytic profiles similar to that of prophage-free IL6288 (**Figure 4C**, data shown for IL6351). The unusual autolytic profiles of the non-permeabilized bIL285-free *L. lactis* cells suggest that bIL285 encodes protein(s) that protect *L. lactis* IL1403 cells from autolysis under the non-growth conditions, In this, *L. lactis* may be similar to *Streptococcus pneumonia*, in which it was recently shown that the presence of a phage-like element correlated with a delay in autolysis (DeBardeleben et al., 2014). This inhibition of autolysis was not attributed to alterations in the amount or activity of the major autolysin LytA, but was instead explained by increased cross-linking within the cell wall.

These results suggest that bIL285-encoded protein(s) may change the autolytic properties of the host by modifying the cellular membrane rather than through interfering with the activity of autolysins. The influence of temperature on this phenomenon is of special interest and may have implications for the industrial and therapeutic applications.

### Prophages Interfere With the Growth Rate and Survival of *L. lactis* Cells

The impact of prophages on bacterial fitness is not obvious. Some prophage-free derivative strains exhibit improved fitness compared to parental strains under conditions that trigger prophage induction, but in other cases prophages can be beneficial for their hosts (Wang et al., 2010; Verghese et al., 2011; Baumgart et al., 2013; Wiles et al., 2013; Martínez-García et al., 2014). Most research conducted to date has focused on the roles of resident prophages and prophage-like elements in *E. coli* and different bacterial pathogens, but less information is available for lysogenic bacteria from other ecological systems (Wang et al., 2010; Casas and Maloy, 2011; Verghese et al., 2011; Fortier and Sekulovic, 2013; Tang et al., 2013).

**FIGURE 5:**
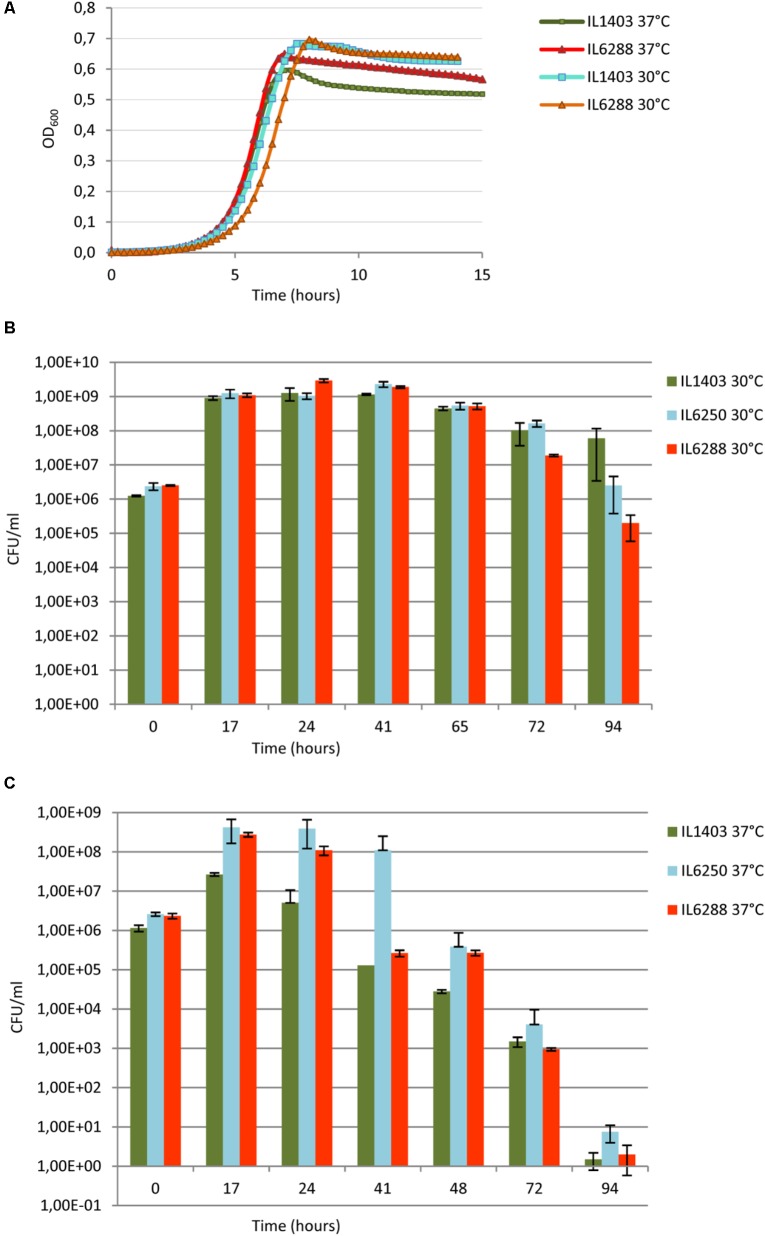
Growth and survival of *L. lactis* IL1403 and its derivative strains at 30°C and 37°C. **(A)** For growth experiments, non-aerated cultures of strains IL1403 and IL6288 were incubated in M17glu medium at the given temperature in a multimode microplate reader (Synergy 2, BioTek Instruments, Inc). OD_600_ was measured at 15-min intervals for the indicated time; cultures were gently agitated prior to sampling. Each experiment included four independent cultures of each strain. For each strain, the mean values of three independent cultures, analyzed simultaneously, are plotted, and the SDs were ≤ 10%. Growth curves of each strain were established at least four times. Survival experiments were performed in static growth conditions at 30°C **(B)** and 37°C **(C)**. Cultures of IL1403, IL6250 (bIL285-, bIL286-, bIL309-free), and prophage-free IL6288 were incubated in M17glu medium at the indicated temperature. Cell survival was monitored by counting CFUs after plating bacterial cultures on M17glu agar. Colonies were counted after 36 h of incubation at 30°C. Each data point (CFUs/ml) is the mean of three counts.

To address the overall effect of the resident prophages on the fitness of *L. lactis* IL1403, we first investigated the growth rate of lysogenic and prophage-free strains at 30°C and at 37°C in rich M17 medium (see the Section “Materials and Methods”). At 30°C, no significant difference was observed between the growth rates of the parental and the prophage-free derivative strains within the time of our experiment (∼15 h) (**Figure 5A**). At 37°C, instead, both strains grew at a uniform rate, but the prophage-free IL6288 strain reached and maintained a plateau of stationary growth at a higher OD than that of the parental strain (**Figure 5A**). These results indicate that the prophages present in *L. lactis* IL1403 do not play an important role during exponential growth under the standard laboratory conditions, but the prophage-free derivative has a growth advantage at the increased incubation temperature. The decrease in growth observed for the parental strain was not caused by prophage induction, as high temperature (37°C) does not provoke the excision of phage DNA (Chopin et al., 2001; Ho et al., 2016).

Next, we examined the effect of resident prophages on the survival of host cells during stationary-phase growth (**Figures 5B,C**). Long-term viability of lactococcal strains is a crucial characteristic for their use in fermentation and storage of dairy foods (Stanton et al., 2005; Bron and Kleerebezem, 2011). Both strains IL1403 and IL6288 showed a rapid decrease in viability during growth at 37°C (**Figure 5C**). In contrast, the survival of IL1403 after 75 h of growth at 30°C was higher than that of its prophage-free derivative (**Figure 5B**). Both strains entered into the stationary phase at the same time, but IL1403 retained at least 90% of its viability for approximately 50 h after reaching the stationary phase, while the viability of the prophage-free strain decreased over that time. To examine the contribution of individual prophages to the survival of the host cells, we then estimated the viability of the intermediate prophage-free derivative strains. The viability of the IL6250 strain carrying only small prophages during the stationary-phase growth at 30°C was intermediate between the parental strain and its prophage-free derivative (**Figure 5B**).

Our results demonstrate that prophage genes improve the stationary-phase survival of lysogenic *L. lactis* strains at a temperature appropriate for growth. Indeed, this type of conditional selection was suggested to contribute to the stabilization of the domesticated prophage genomes (Bobay et al., 2014). Thus, prophage genes could represent an important selective advantage for *L. lactis* strains during the fermentation process.

### Prophages Adversely Affect Aerobic and Heme-Activated Respiration Growth of *L. lactis*

Survival of *L. lactis* in aerobic environments is generally poor, as oxygen induces DNA, protein and membrane damage (Duwat et al., 2001; Rezaïki et al., 2004). However, *L. lactis* growth can be greatly improved under these conditions if there is a supply of heme, an iron-containing porphyrin essential for activation of the respiration electron transfer chain. Compared with aerobic growth, respiratory growth increases lactococcal biomass and long-term survival (Rezaïki et al., 2004). Because of this, heme-activated respiration conditions have been already used for dairy starter production and may open new avenues for the use of *L. lactis* in health-related contexts (Teusink and Smid, 2006; Pedersen et al., 2012). However, free heme causes toxicity at high concentrations due to its ability to generate reactive oxygen species (Lechardeur et al., 2011).

**FIGURE 6:**
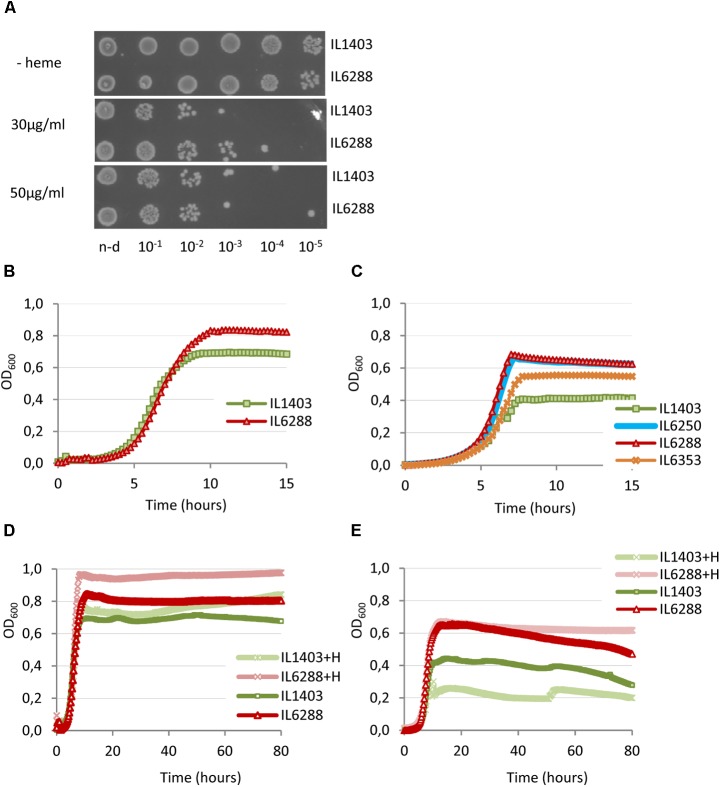
Effect of heme on the growth of *L. lactis* IL1403 and its prophage-free derivative strain IL6288. For the assay of heme-induced toxicity **(A)**, autoclaved heme was added to static cultures of *L. lactis* IL1403 and IL6288 at an OD_600_ of 0.6 at the indicated concentrations. Cultures were incubated at 30°C under non-aerated conditions. After 1 h, viable cell numbers were determined by spotting indicated serial dilutions of the bacterial cultures onto M17glu agar. Images were acquired with a ChemiDoc MP system after 36 h of incubation at 30°C. This experiment was performed twice; the results of a representative experiment are shown. For the characterization of growth under aerated conditions **(B–E)**, cultures of IL1403 and its derivative strains were incubated in M17glu medium with (+H) or without heme in a multimode microplate reader (Synergy 2, BioTek Instruments, Inc) with constant shaking at 30°C **(B,D)** or 37°C **(C,E)**. Each experiment included four independent cultures of each strain. For each strain, the mean values of three independent cultures, analyzed simultaneously, are plotted. Growth curves of each strain were established three times. The results presented are of a representative experiment.

Considering the importance of this aspect of lactococcal physiology, we wanted to investigate how prophage content influences the resistance of *L. lactis* IL1403 to heme and its survival under aeration and/or respiration conditions. First, we determined whether resident prophages influence the tolerance of the bacterial host to heme (Materials and Methods). As shown in **Figure 6A**, there were only slight differences between strains IL1403 and IL6288 in sensitivity to treatment with heme, which was performed under static conditions of growth at 30°C. This suggests that resident prophages have little or no impact on the regulation of heme uptake and degradation, which are known to determine heme tolerance in bacteria (Lechardeur et al., 2011).

We next compared the growth of the parental strain and its derivatives under aerobic conditions at 30°C and 37°C (see the Section “Materials and Methods”). For all strains, the negative effects of oxygen on cell growth were more apparent at 37°C than at 30°C. As shown in **Figures 6B,C**, the growth period of prophage-free strain IL6288 was extended compared to that of the parental strain, so that IL6288 cultures entered into the stationary phase at a higher optical density than those of IL1403. In general, the growth kinetics of the prophage-free strain were similar to those of intermediate strains that carried only the three small prophages (IL6250) or strains with the small prophages and either bIL309 or bIL286 (**Figure 6C**; data shown for IL6250 grown at 37°C). Instead, the strains that carried the three small prophages together with bIL285 (strain IL6353) or with bIL309 and bIL286 (strain IL1946) showed intermediate growth profiles (**Figure 6C**; data shown for IL6353 grown at 37°C).

It has been shown that the cleavage of the lactococcal phage repressors by RecA results in prophage induction (Chopin et al., 2001). In *L. lactis*, oxygen provokes DNA-damage and an increase of RecA activity (Rezaïki et al., 2004). Thus, the oxidative stress may stimulate induction of the resident prophages, which in turn may negatively affect bacterial growth. In addition, negative effect of the increased temperature in the conditions stimulating DNA damage might be due to the thermosensitive nature of the double-strand break repair enzyme, RexAB, responsible for the main recombination-mediated DNA repair pathway in lactococci (El Karoui et al., 1998).

Because DNA damage of *L. lactis* cells under respiration conditions is reduced by the presence of heme (Rezaïki et al., 2004), we next examined the effect of heme addition on the growth of two *L. lactis* strains in aerobic conditions (see the Section “Materials and Methods”). As shown in **Figures 6D,E**, the growth of the prophage-free strain was improved by the addition of heme at both 30°C and at 37°C. Conversely, the growth of IL1403 was improved at 30°C but was weakened at 37°C. This suggests that heme is more toxic to cells grown under aeration conditions at increased temperature. Moreover, this effect correlates with the presence of prophages.

These data demonstrate that deletion of the resident prophages from strain IL1403 positively affects the aerobic growth of lactococcal cells and significantly increases the positive effect of heme-activated respiration.

### Prophage-Free Strain Has Improved Survival in the Digestive Tract of Germfree Mice

The majority of *L. lactis* strains do not survive the passage through the digestive tract. However, this bacterium’s high potential for therapeutic use has stimulated the search for factors that increase its ability to persist in the gastrointestinal environment (Vesa et al., 2000; Kimoto et al., 2003). Under these conditions, bacterial physiology may be affected in a number of ways. For example, the different physicochemical stressors in the digestive tract (e.g., nitric oxide or bile salts) might activate the bacterial SOS response and subsequently, the induction of prophages. Even prior to induction, though, prophage-encoded functions might modify the host’s PG and/or cell membrane, weaken the cell wall, and increase the sensitivity of lysogenic bacteria to lysozyme and other host defense factors (see above).

**FIGURE 7:**
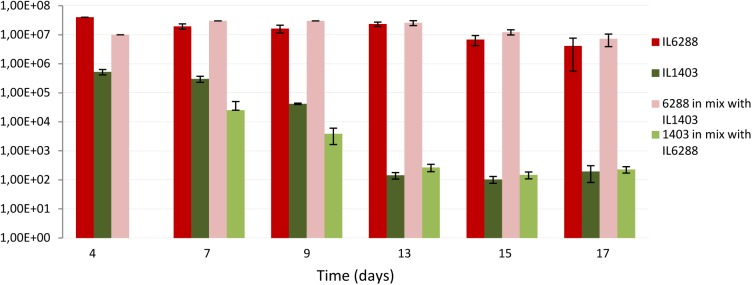
Survival of *L. lactis* IL1403 and its prophage-free derivative strain, IL6288 after passage through the gastrointestinal tract of germ-free mice. Over 3 consecutive days, mice were orally gavaged with 16% glycerol solution in PBS buffer containing either 10^9^ CFUs of strain IL1403, or 10^9^ CFUs of strain IL6288, or the mixture of 10^9^ CFUs of strain IL1403:Em^R^ plus 10^9^ CFU of strain IL6288:Cm^R^. Each strain was recovered from mouse feces at the indicated time points; cell survival was determined by counting CFUs on M17glu agar plates after 36 h of incubation at 30°C. Each data point (CFUs/ml) is the mean of at least three measurements.

To evaluate the impact of the resident prophages on lactococcal survival and/or colonization in the digestive tract, we used a germfree mouse model. Specifically, we determined the viability of *L. lactis* IL1403 and IL6288 cells after oral gavages of either pure (IL1403, IL6288) or mixed (50:50 IL1403:IL6288) bacterial cultures (see the Section “Materials and Methods”). After passage through the stressful gastrointestinal conditions, strain IL6288 demonstrated significantly enhanced survival compared to the parent strain. As shown in **Figure 7**, the population of IL1403 cells decreased considerably during the experimental period (from approximately 10^5^ CFUs/g feces at the 4th day after administration to 10^2^ CFU/g feces at the 17th day, either in pure IL1403 culture or in mixed culture with IL6288). This is consistent with previously published results demonstrating the poor survival of lactococcal strains in the digestive tract (Drouault et al., 1999; Vesa et al., 2000; Kimoto et al., 2003). In contrast, the population of IL6288 cells was relatively stable, remaining at approximately 10^7^ to 10^6^ CFUs/g feces both in the pure and the mixed culture during the experimental period (17 days).

We suggest that, the increased resistance to lysozyme (see above) can be an important factor determining the survival of IL6288 cells through the digestive tract of mice. On the other hand, the repression system(s) of IL1403 prophages may be rather sensitive to some specific gastrointestinal stresses. Thus, prophage induction may also contribute negatively to the survival of the lysogenic strain.

These data indicate that the deletion of the resident prophages was a decisive factor in the ability of *L. lactis* cells to survive within the gastrointestinal environment. This finding may have major implications for the use of *L. lactis* strains as biotherapeutic agents, because prophage-free derivatives may be able to function more effectively as vaccines and/or probiotic strains.

## Conclusion

Our results reveal the significant contribution that resident prophages make to diverse aspects of *L. lactis* cell physiology, many of which are relevant to the use of *L. lactis* for industrial and health applications. They also indicate that a resident prophage, alone or in combination with others, shapes the cellular phenotype and influences its plasticity in response to the environment. Under experimental conditions that were comparable to those of industrial fermentation, the lactococcal prophages were mostly beneficial for host survival. However, the presence of prophages had clear adverse effects when *L. lactis* was exposed to the conditions of the gastrointestinal environment. Our construction of a prophage-free strain of *L. lactis* opens new avenues for both fundamental and applied studies of different aspects of lactoccoccal cell behavior, including the response to environmental stresses and bacteria-phage and bacteria-host interactions in a “pure” bacterial genetic background. As *L. lactis* continues to grow in economic importance, this work can assist in the rational selection of strains for both the industrial manufacturing of fermented dairy products and therapeutic applications for human and animal health.

## Author Contributions

EB, FC, and PL designed the experiments. AA and EB performed all the major experiments. AA and FC carried out the animal experiment. EB wrote the manuscript. All the authors approved the manuscript for publication.

## Conflict of Interest Statement

The authors declare that the research was conducted in the absence of any commercial or financial relationships that could be construed as a potential conflict of interest.

## Footnotes

1 http://www.ncbi.nlm.nih.gov/genome/genomes/156
