# Supplementary material for: Characterization of a Prophage-Free Derivative Strain of Lactococcus lactis ssp. lactis IL1403 Reveals the Importance of Prophages for Phenotypic Plasticity of the Host
Source: Front Microbiol. 2018 Aug 31;9:2032. doi: 10.3389/fmicb.2018.02032 (PMC6127208; doi:10.3389/fmicb.2018.02032)
Supplement: Supplementary file 1 [file Table_1.DOCX]

Table S1. Oligonucleotides used for strains construction

| Oligonucleotide | Sequence (5’->3’)*^a^* |
| --- | --- |
| AA07 | **tcccccggggga**gtgaagccgaagctgttgcag |
| AA08 | **tcccccggggga**gctggcaatgagtgtttatg |
| AA26 | ctgttcggcctgagccttg |
| AA27 | ccacggcaacctcaagttc |
| AA28 | gggatcaggataatcatccgc |
| AA29 | gaacctatcagaattgatgc |
| AA30 | **tcccccggggga**caatgaagagagcagctgtcc |
| AA31 | **tcccccggggga**caagtctagcaatcactgtg |
| AA32 | **tcccccggggga**ggcatagcgattaataatgcgg |
| AA33 | **tcccccggggga**gtcagggctattgatgatttc |
| AA34 | **tcccccggggga**gccttgattggcgaagctag |
| AA35 | **tcccccggggga**gttctgacttcttactctctgc |
| AA36 | ggcgatggacaaagggttg |
| AA37 | tgtaaaacgacggccagt |
| AA38 | ggaaacagctatgaccatg |
| AA39 | ggaaatcagagagaagatc |
| AA40 | gcaagatggccattgctaag |
| AA41 | ctctgtgtcaacgtcaacc |
| AA42 | cagaactcccaatgctcattg |
| AA43 | gcgaatcaaattgatttagcc |
| AA44 | gtggttgccattgttgaag |
| AA45 | ggtaactcgtagaatgtacg |
| AA46 | gtgtaccagccttgcccc |
| AA47 | **tcccccggggga**ggcggagcatgaattcatc |
| AA53 | **tcccccggggga**gtatccatggcggaatggtc |
| AA56 | gtagggcataaggatggcgg |
| AA57 | gaaggtcaacgtggtcttc |
| AA60 | gttgataagctggtattc |
| MCC31 | gtgagagaattacaacggag |
| MCC32 | gctcggtcatagtagtttg |
| MCC35 | gacacatacagccaccttg |
| MCC44 | ctcagaagttgcaagtcg |

*a*: *Sma*I and *Kpn*I restriction sites are shown in bold.
